# Urinary metabolites associate with the presence of diabetic kidney disease in type 2 diabetes and mediate the effect of inflammation on kidney complication

**DOI:** 10.1007/s00592-023-02094-z

**Published:** 2023-05-15

**Authors:** Caifeng Shi, Yemeng Wan, Aiqin He, Xiaomei Wu, Xinjia Shen, Xueting Zhu, Junwei Yang, Yang Zhou

**Affiliations:** Center for Kidney Disease, Second Affiliated Hospital of Nanjing Medical University, No. 262 N Zhongshan Road, Nanjing, 210003 Jiangsu China

**Keywords:** Metabolite, Inflammation, Kidney

## Abstract

**Aims:**

Diabetic kidney disease (DKD) is the one of the leading causes of end-stage kidney disease. Unraveling novel biomarker signatures capable to identify patients with DKD is favorable for tackle the burden. Here, we investigated the possible association between urinary metabolites and the presence of DKD in type 2 diabetes (T2D), and further, whether the associated metabolites improve discrimination of DKD and mediate the effect of inflammation on kidney involvement was evaluated.

**Methods:**

Two independent cohorts comprising 192 individuals (92 DKD) were analyzed. Urinary metabolites were analyzed by targeted metabolome profiling  and inflammatory cytokine IL-18 were measured by ELISA. Differentially expressed metabolites were selected and mediation analysis was carried out.

**Results:**

Seven potential metabolite biomarkers (i.e., S-Adenosyl-L-homocysteine, propionic acid, oxoadipic acid, leucine, isovaleric acid, isobutyric acid, and indole-3-carboxylic acid) were identified using the discovery and validation design. In the pooled analysis, propionic acid, oxoadipic acid, leucine, isovaleric acid, isobutyric acid, and indole-3-carboxylic acid were markedly and independently associated with DKD. The composite index of 7 potential metabolite biomarkers (CMI) mediated 32.99% of the significant association between the inflammatory IL-18 and DKD. Adding the metabolite biomarkers improved the discrimination of DKD.

**Conclusions:**

In T2D, several associated urinary metabolites were identified to improve the prediction of DKD. Whether interventions aimed at reducing CMI also reduce the risk of DKD especially in patients with high IL-18 needs further investigations.

**Supplementary Information:**

The online version contains supplementary material available at 10.1007/s00592-023-02094-z.

## Introduction

Diabetic kidney disease (DKD) is the major complication of type 2 diabetes (T2D) [1] and the one of the leading causes of end-stage kidney disease (ESRD) [2, 3] that creates tremendous medical care costs. Unraveling novel biomarker signatures capable of accurate identifying patients with DKD for more specific and aggressive intervention is favorable and economical for solve the burden.

In recent decades, studies have investigated the role of circulating metabolite biomarkers in predicting the incidence and outcome of T2D [4–7], and several have focused on the development and progression of DKD [8–15]. However, few have addressed the association of urinary metabolites with DKD [16–18]. In detail, urinary metabolites associated with DKD are mostly amino acids [16–18], tricarboxylic acid (TCA) cycle intermediates [17, 18]. Unfortunately, the untargeted metabolome profiling studies on future DKD progression have been carried out in subgroups of chronic kidney disease cohort, and therefore, the metabolite signatures may not be optimal [17]. The screening and validation study on differentially expressed metabolites between diabetes with or without kidney involvement, however, initially identified the candidate metabolites in healthy control and DKD groups [18].

Here we investigated the potential association between a variety of urinary metabolites and presence of DKD in individuals with T2D. Some metabolites were unraveled and validated as independently and robustly associated. Whether these metabolites contribute to the effect of inflammatory cytokine on DKD were also explored. We further investigated whether a total of metabolites and inflammatory cytokine improved the diagnosis of DKD.

## Research design and methods

### Participants

Two cohorts of patients with T2D from Nanjing, east China, were analyzed. T2D was diagnosed according to American Diabetes Association 2018 criteria. The discovery cohort included 77 patients, and the validation cohort included 115 patients. DKD was diagnosed as urinary albumin-to-creatinine (ACR) ≥ 30 mg/g or estimated glomerular filtration rate (eGFR) < 60 ml/min/1.73 m^2^. eGFR was calculated using the chronic kidney disease epidemiology collaboration equation (CKD-EPI equation).

### Clinical examinations

Blood pressure (BP), height, and weight were measured by a trained nurse. BP was assessed using an arm electronic sphygmomanometer. Two separate measurements were taken for systolic and diastolic BP, and the average was recorded. In cases where there was a BP difference of 10 mmHg for systolic BP or 5 mmHg for diastolic BP, a third measurement was taken. The two closest BP measurements were then averaged and recorded. Hypertension was defined as systolic BP > 140 mmHg or diastolic BP > 90 mmHg or on antihypertension medication. Weight was measured using a calibrated digital weight scale (Omron Karada Scan Body Composition Scale HBF-375, Osaka, Japan), while height was measured using a wall-mounted and adjustable tape. Body mass index (BMI) was then calculated using weight (kilogram) divided by the square of height (meter). The biochemical parameters including albumin, hemoglobin A1c (HbA1c), fasting blood glucose (FBG), total cholesterol (TC), triglycerides (TG), high-density lipoprotein (HDL), low-density lipoprotein (LDL), serum creatinine, uric acid and urinary ACR were measured. All participants were categorized as smokers or non-smokers, and alcohol consumption or not based on their responses in a standard questionnaire. Information on drug therapy was obtained from electronic medical record of each participant.

### Measurement of metabolite and IL-18

Blood and morning urine samples at baseline were stored at −80 °C until analysis. We performed the metabolomics analysis by Q300 Kit (Metabo-Profile, Shanghai, China). Urinary samples were treated, and the metabolites were quantitated using an ultraperformance liquid chromatography coupled to tandem mass spectrometry (UPLC-MS/MS) system (ACQUITY UPLC-Xevo TQ-S, Waters Corp., Milford, MA, USA) by Metabo-Profile Biotechnology (Shanghai) Co., Ltd.[19]. Serum and urinary IL-18 were measured twice for each individual using sandwich ELISA kits (DY318-05, R&D Systems) [20].

### Statistical analysis

Metabolite data were processed using iMAP platform (v1.0; Metabo-Profile, Shanghai, China). Principal component analysis (PCA) was performed. The V plot integrates the fold change and *P* values. The Z-score indicates standard deviations concerning the mean of the control group. Variable importance in projection (VIP) was obtained based on the orthogonal partial least squares discriminant analysis (OPLS-DA) model. Differentially expressed metabolites (DEMs) were selected with VIP ≥ 1 and *P* value < 0.05.

Baseline characteristics of the enrolled subjects were reported as mean ± SD or median (interquartile range) for continuous variables and frequency (percentage) for categorical variables. Differences between groups in continuous variables were tested by *t* test or Wilcoxon signed-rank test after checking for normality and Chi-squared test for class variables. IL-18 concentrations were logarithmically transformed because of skewed distribution. The associations between seven potential metabolic markers as well as IL-18 and DKD were tested using logistic regression models. To facilitate comparison of results, all ORs were reported per SD units. Mediation analysis was carried out for causal interpretation and exposure–mediator interactions detection [21]. A *P* value < 0.05 was considered statistically significant. All analyses were performed using SPSS 25 (process procedure for SPSS version 3.4) and R software.

## Results

### Metabolites and DKD

Clinical features of patients from discovery and validation cohorts are summarized in Table 1. In the two independent cohorts, the presence of DKD was 49.35% (38/77) and 46.96% (54/115), respectively.

**Table 1:** Clinical characteristics of the two independent study cohorts

Features	Discovery cohort (*n* = 77)			Validation cohort (*n* = 115)		
	T2D (*n* = 39)	DKD (*n* = 38)	*P*	T2D (*n* = 61)	DKD (*n* = 54)	*P*
Male (*n*, %)	24 (61.54%)	28 (73.68%)	0.255	39 (63.93%)	39 (72.22%)	0.342
Age (years)	54.00 (44.00, 60.00)	52.50 (41.25, 59.50)	0.931	57.00 (53.00, 60.00)	58.00 (50.75, 62.00)	0.700
Alcohol (n, %)	13 (33.33%)	16 (42.11%)	0.427	23 (37.70%)	24 (44.44%)	0.463
Smoke (n, %)	18 (46.15%)	21 (55.26%)	0.424	31 (50.82%)	25 (46.30%)	0.628
BMI (kg/m^2^)	26.15 ± 3.61	25.18 ± 3.17	0.227	24.08 ± 2.61	25.82 ± 3.87	0.006
SBP (mmHg)	130.44 ± 13.96	140.41 ± 16.20	0.005	132.18 ± 15.78	141.80 ± 19.81	0.005
DBP (mmHg)	83.18 ± 10.02	89.27 ± 12.73	0.023	80.95 ± 9.39	88.15 ± 9.98	< 0.001
Hypertension (n, %)	13 (33.33%)	15 (39.47%)	0.575	27 (44.26%)	34 (62.96%)	0.045
Diabetes duration (months)	11.00 (4.00, 34.00)	22.50 (6.75, 54.00)	0.181	135.00 (89.50, 194.00)	126.50 (58.25, 196.25)	0.799
Albumin (g/L)	48.50 (45.70, 50.00)	47.60 (43.55, 49.55)	0.109	44.90 ± 3.87	43.40 ± 5.30	0.085
FBG (mmol/L)	9.21 (7.56, 11.26)	8.19 (6.46, 11.12)	0.349	8.10 (6.75,11.03)	8.88 (6.80, 11.88)	0.560
HbA1c (%)	7.65 (6.60, 9.93)	8.00 (6.88, 9.95)	0.755	8.30 (6.95,10.20)	8.70 (7.10, 10.20)	0.678
TC (mmol/L)	4.54 ± 1.13	4.98 ± 1.38	0.130	4.50 (3.68,5.00)	4.52 (3.92, 5.17)	0.407
TG (mmol/L)	1.49 (1.12, 2.39)	2.11 (1.33, 3.27)	0.077	1.42 (0.89, 1.95)	1.65 (1.12, 2.52)	0.055
HDL (mmol/L)	1.13 (0.95, 1.29)	0.99 (0.90, 1.30)	0.499	1.08 (0.96, 1.34)	1.07 (0.92, 1.25)	0.724
LDL (mmol/L)	3.13 (2.38, 3.98)	3.03 (2.25, 3.88)	0.689	2.84 (2.30, 3.19)	2.71 (2.28, 3.25)	0.935
Uric acid (μmol/l)	299.00 (276.00, 376.00)	330.50 (263.00, 402.70)	0.436	279.00 (223.50, 330.50)	339.50 (300.50, 403.00)	< 0.001
Serum creatinine (μmol/l)	64.40 (53.10, 80.90)	68.25 (54.43, 84.38)	0.548	61.00 (51.95, 70.90)	78.90 (61.60, 106.73)	< 0.001
eGFR (ml/min/1.73m^2^)	103.85 (87.60, 114.54)	103.12 (88.59, 113.70)	0.799	102.60 (96.59, 108.75)	91.72 (64.96, 105.81)	< 0.001
ACR (mg/g)	8.60 (5.70, 13.70)	69.05 (37.18, 275.30)	< 0.001	8.10 (4.80, 13.95)	195.45 (64.76, 695.23)	< 0.001
ACE inhibitor or ARB (n, %)	2 (5.13%)	6 (15.79%)	0.154	5 (8.20%)	15 (27.78%)	0.006
SGLT2 inhibitor (n, %)	5 (12.82%)	15 (39.47%)	0.008	8 (13.11%)	16 (29.63%)	0.030

Continuous variables were reported as mean ± SD, whereas skewed variables as median (interquartile range). Categorical variables were presented as total frequencies and percentages

ACE, angiotensin-converting enzyme; ACR, albumin-to-creatinine ratio; ARB, angiotensin II receptor blocker; BMI, body mass index; DBP, diastolic blood pressure; DKD, diabetic kidney disease; eGFR, estimated glomerular filtration rate; FBG, fasting blood glucose; HbA1c, hemoglobin A1c; HDL, high-density lipoprotein; LDL, low-density lipoprotein; SBP, systolic blood pressure; SGLT2, sodium glucose co-transporter 2; T2D: type 2 diabetes. TC, total cholesterol; TG, triglycerides

The absolute concentrations of 160 metabolites were targeted measured using UPLC-MS/MS system in a total of 192 participants (supplemental Table 1 [discovery cohort] and supplemental Table 2 [validation cohort]).

Using both VIP ≥ 1 in multi-dimensional statistics and *P* < 0.05, |log_2_FC|≥ 0 in univariate statistics as the threshold value, 64 out of the 160 analyzed metabolites in the discovery cohort were selected as differential metabolites, with their value shown in supplemental Table 3.

We carried out Boruta analysis to find out candidate biomarkers from differential metabolites. Plot of feature selection result by Boruta is shown in supplemental Fig. 1. Among the 64 differential metabolites identified in discovery cohort, 19 metabolites labeled as “Confirmed” (Green box in supplemental Fig. 1) in the plot could serve as candidate biomarker.

In the totally independent validation cohort, 31 differential metabolites out of the 160 analyzed metabolites matched the threshold value are shown in supplemental Table 4. Venn plot of 7 potential biomarkers (i.e., S-Adenosyl-L-homocysteine [SAH], propionic acid, oxoadipic acid, leucine, isovaleric acid, isobutyric acid, and indole-3-carboxylic acid) from the 19 candidate biomarkers in discovery cohort and 31 differential metabolites of validation cohort are shown in supplemental Fig. 2.

**Fig. 1:**
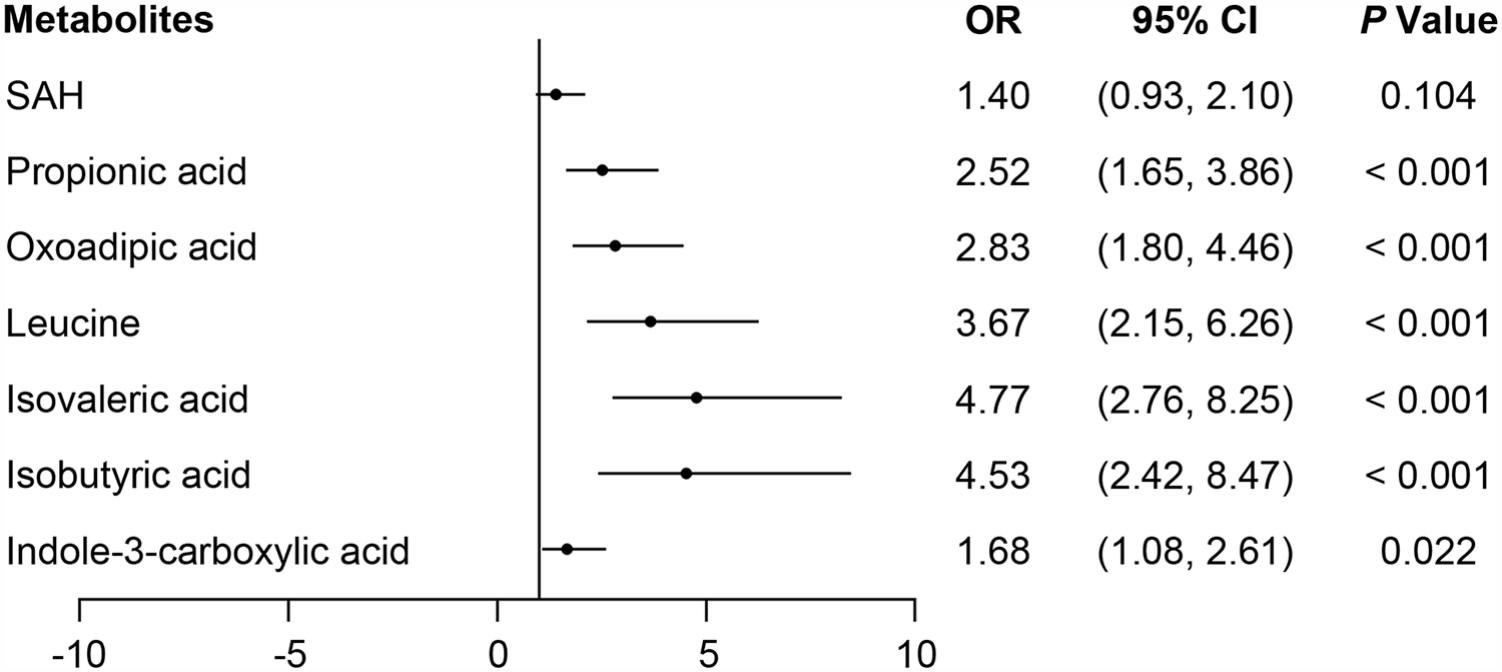
Independent associations between metabolite biomarkers and DKD. Odds ratio (OR) and 95% confidence intervals (CIs) for independent associations between 7 potential metabolite biomarkers and DKD in the pooled cohort. OR (per 1SD increase in the logarithmically transformed of each metabolite concentration) was estimated in logistic regression models, adjusting for age, gender, BMI, hypertension, diabetes duration, HbA1c, Albumin, total cholesterol, and eGFR

The seven validated associations were then analyzed using data from the two independent cohorts comprising a total of 192 individuals and 92 DKD (Fig. 1), propionic acid (OR 2.52, 95% CI 1.65–3.86), oxoadipic acid (OR 2.83, 95% CI 1.80–4.46), leucine (OR 3.67, 95% CI 2.15–6.26), isovaleric acid (OR 4.77, 95% CI 2.76–8.25), isobutyric acid (OR 4.53, 95% CI 2.42–8.47), and indole-3-carboxylic acid (OR 1.68, 95% CI 1.08–2.61), which were markedly associated with the presence of DKD (per 1 SD increase in the logarithmically transformed of each metabolite concentration) with no difference between each gender (all *P* > 0.05).

### IL-18, metabolites, and DKD

In the past decade, investigations have elucidated specific DKD progression processes, including inflammatory and metabolic [22]. Receptors of the innate immune system may modulate the aberrant metabolic adaptations during kidney disease—that is immunometabolism [23].

The association between IL-18 related to innate inflammation and both metabolites and DKD was then investigated. Serum and urinary IL-18 as well as the composite index of 7 potential metabolite biomarkers (CMI) are shown in supplemental Table 5.

As a matter of fact, urinary IL-18 was associated with CMI (supplemental Table 6). Urinary IL-18 was also associated with the presence of DKD in the fully adjusted model (Table 2, left panel). Of note, the association with DKD of urinary IL-18 was no longer significant when adding CMI into the model (Table 2, right panel).

**Table 2:** Univariable associations between IL-18 and DKD in pooled sample (*n* = 192; 92 DKD)

	Association with DKD		Association with DKD adjusted also for CMI	
	OR (95% CI)	*P*	OR (95% CI)	*P*
Serum IL-18	0.96 (0.67, 1.35)	0.796	0.96 (0.60, 1.52)	0.849
Urinary IL-18	1.99 (1.32, 3.00)	0.001	1.31 (0.76, 2.27)	0.334

ORs were estimated in logistic regression models, adjusting for age, gender, hypertension, diabetes duration, BMI, HbA1c, total cholesterol, albumin, and eGFR. ORs reflect the risk per 1SD increase in the logarithmically transformed of IL-18 concentration

CMI, composite index of 7 potential metabolite biomarkers; DKD, diabetic kidney disease; IL: interleukin

**Fig. 2:**
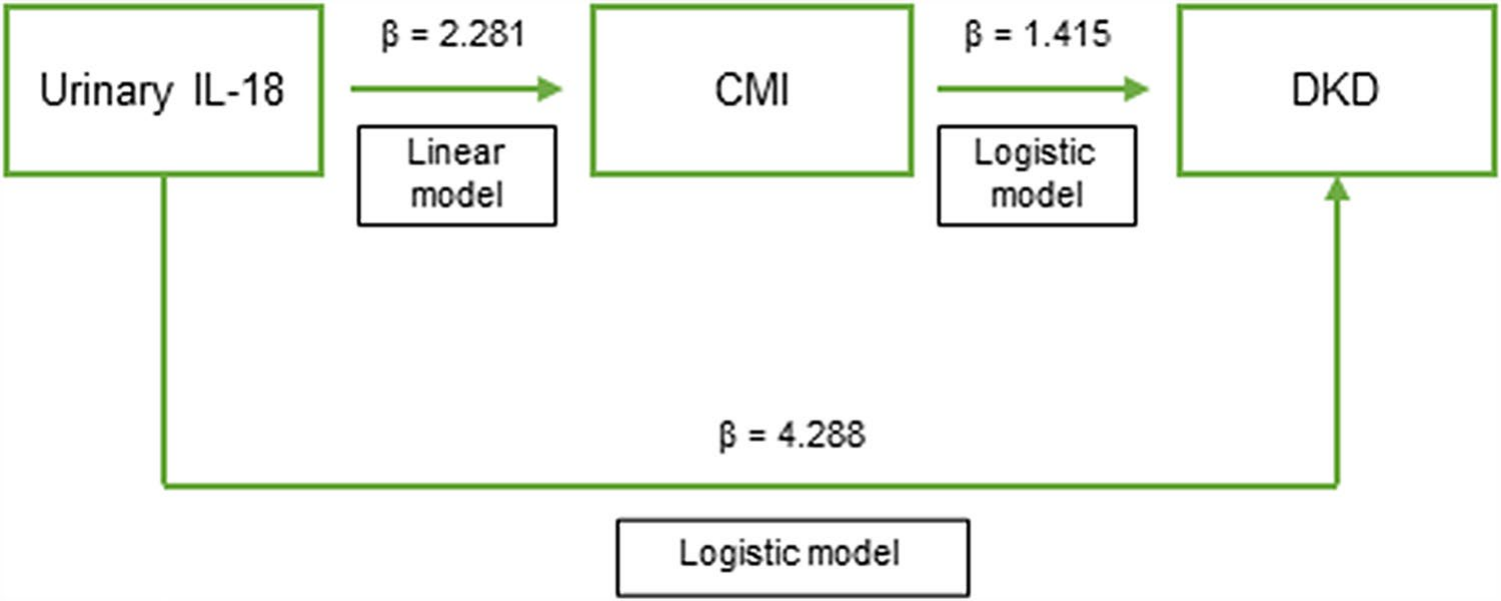
Mediation model showing the role of CMI on the association between urinary IL-18 and DKD in the pooled sample. Mediation analysis was carried out in a fully adjusted model, comprising age, gender, hypertension, diabetes duration, BMI, HbA1c, total cholesterol, albumin, and eGFR. *β* = standardized coefficient of regression. The total effect of IL-18 (*β* = 4.288) on the outcome partly passes through metabolites (*β* of the CMI mediated effect of IL-18 = 1.415). The proportion explained by the CMI is equal to 32.99% (i.e., 1.415/4.288)

Consistently, mediation analysis further indicated a marked and non-trivial proportion (i.e., 32.99%) of the association between IL-18 and DKD were through CMI (Fig. 2, Supplemental Table 7).

### Metabolites and prediction of DKD

In the two independent cohort and the pooled sample, discrimination ability of CMI was 91.1% (95% CI 84.6%–97.6%), 79.0% (95% CI 70.8%–87.2%), and 82.0% (95% CI 76.2%–87.9%), respectively (Table 3). The effect of adding CMI on top of urinary IL-18 showed a significant improvement [i.e., 91.2% (95% CI 84.8%–97.7%) in discovery cohort, 81.4% (95% CI 73.7%–89.2%) in validation cohort and 83.4% (95% CI 77.8%–89.0%) in pooled sample] (Table 3).

**Table 3:** Area under the receiver operator characteristics curve categorized by metabolites and urinary IL-18

	Discovery cohort (*n* = 77)			Validation cohort (*n* = 115)			Pooled sample (*n* = 192)		
	AUC (95% CI)	Specificity	Sensitivity	AUC (95% CI)	Specificity	Sensitivity	AUC (95% CI)	Specificity	Sensitivity
Propionic acid	76.3 (65.1, 87.6)	71.8	81.6	63.9 (53.6, 74.2)	62.3	72.2	68.0 (60.3, 75.7)	69.0	65.9
Oxoadipic acid	70.0 (58.4, 81.6)	30.8	100.0	64.8 (54.6, 75.1)	73.8	55.6	66.9 (59.3, 74.5)	78.0	49.5
Leucine	80.4 (70.6, 90.2)	76.9	73.7	62.7 (52.2, 73.3)	62.3	68.5	69.4 (61.7, 77.0)	62.0	75.8
Isovaleric acid	83.4 (74.2, 92.6)	84.6	71.1	69.7 (60.1, 79.4)	82.0	50.0	76.5 (69.7, 83.2)	84.0	58.2
Isobutyric acid	82.9 (73.6, 92.1)	74.4	78.9	72.8 (63.7, 82.0)	72.1	64.8	77.1 (70.5, 83.7)	70.0	73.6
Indole-3-carboxylic acid	68.8 (56.7, 80.8)	82.1	57.9	64.9 (54.8, 75.0)	72.1	57.4	66.1 (58.3, 73.8)	76.0	57.1
SAH	77.7 (67.1, 88.3)	89.7	63.2	65.3 (54.8, 75.7)	82.0	51.9	69.5 (61.8, 77.2)	79.0	60.4
CMI	91.1 (84.6, 97.6)	89.7	81.6	79.0 (70.8, 87.2)	73.8	72.2	82.0 (76.2, 87.9)	86.0	65.9
Urinary IL-18	66.3 (53.9, 78.6)	41.0	94.7	67.5 (57.6, 77.3)	77.0	57.4	66.2 (58.6, 73.9)	74.0	50.5
Urinary IL-18 + CMI	91.2 (84.8, 97.7)	84.6	86.8	81.4 (73.7, 89.2)	65.6	90.7	83.4 (77.8, 89.0)	90.0	62.6

AUC, area under the receiver operator characteristics curve; CMI, composite index of 7 potential metabolite biomarkers; IL, interleukin; SAH, S-Adenosyl-L-homocysteine

In summary, our data consistently suggested that levels of urinary metabolites improved the discrimination of DKD in patients with type 2 diabetes.

## Discussion

In this study, we rigorously evaluated the association between 160 metabolites measured through targeted metabolomics and incidence of DKD in 192 people with type 2 diabetes using a discovery and validation design. Seven potential urinary metabolite biomarkers were identified by metabolomics analysis. Among them, propionic acid, oxoadipic acid, leucine, isovaleric acid, isobutyric acid, and indole-3-carboxylic acid were independently associated with DKD in the pooled cohort of patients with T2D. Of note, taking into account of eGFR, a strong indicator of DKD, did not diminish the relationship between metabolites and the presence of DKD. This study suggested that the associations were independent of blood glucose level, BMI, or diabetes duration.

The seven potential metabolite markers met the selection threshold of both multi-dimensional statistics and univariate statistics in both discovery and validation cohorts. Some of the them have been previously reported, and others are newly discovered. Propionic acid, isovaleric acid, and isobutyric acid, all short-chain saturated fatty acid, have been previously associated with the development and progression of kidney damage in diabetes patients [17, 18]. Leucine is one of the essential amino acids in humans and is involved in protein synthesis and many metabolic functions. Increased levels of leucine in both blood and urine were indicative for diabetes [8] and adverse renal outcome [15, 16]. Oxoadipic acid, a key metabolite of the essential amino acids tryptophan [7, 11, 12] and lysine, also an important metabolite between the TCA cycle and lysine biosynthesis, plays a major role on mitochondrial metabolite transport. SAH is an amino acid derivative and an intermediate in the synthesis of cysteine and adenosine, which modulates methylation-dependent reactions and thus reflects methylation status of macromolecules. Not surprisingly, when considered together the seven potential metabolite markers (CMI) showed a good ability in discriminating DKD which may be of great clinical significance.

Inflammation mediates hyperglycemia-induced series of intracellular processes to promote kidney damage in diabetes [24, 25]. Altered intracellular metabolism generates a variety of signalings and molecules to promote the production of pro-inflammatory mediators [25]. Both increased nuclear factor-κB (NF-κB) [22, 26] and activated nucleotide-binding oligomerization domain-like receptors pyrin domain containing 3 (NLRP3) inflammasome induced the expression and activation of interleukins (IL), including IL-18 [27]. Elevated IL-18 has been closely implicated in the development and progression of DKD [28]. In the meantime, higher IL-18 has been described in CKD patients as potential biomarkers [29]. Urine IL-18 was upregulated in response to tubular injury and was associated with adverse kidney events and death [30]. Nevertheless, the ability of IL-18 to distinguish renal function is still controversial [31–33].

Growing evidence has indicated that metabolic and inflammatory profiles of kidney proximal tubule were influenced by various injuries and further alterations occurred with damage progression or repairment [34]. Activation of innate immune sensors in part contributed to the metabolic adaptations of kidney tubules [35]. Therefore, kidney tubule is probably regulated by immunometabolism [23]. Activation of NLRP3 inflammasome during injury appeared to be closely related to metabolism; however, the underlying mechanisms remain unclear [36]. Accordingly, the mediation analysis in this study showed a proportion of 32.99% of the association between IL-18 and DKD were through metabolites. Moreover, adding metabolites on top of urinary IL-18 showed a significant improvement of discrimination of DKD.

In the two independent cohorts, patients with DKD have higher blood pressure but similar blood glucose and lipid levels compared to those without DKD. To date, the only proven primary prevention interventions for the development of DKD are blood glucose and blood pressure control [37]. Intensive lowering of blood glucose has been shown in large randomized studies to delay the onset and progression of DKD in people with diabetes [38, 39]; however, adverse effects including hypoglycemia and mortality were increased among people with kidney evolvement at baseline [40, 41]. Hypertension is a strong risk factor for the development and progression of CKD [42]. Antihypertensive therapy reduces the risk of albuminuria [43], progression to ESRD [44], and cardiovascular events [43]. Lipid management including lifestyle intervention, lipid-lowing, and weight-loss strategies demonstrated reduction in the risk of adverse cardiovascular and kidney events [45]. Besides, a number of cardiovascular risk factors have recently been observed to influence the main clinical outcomes of patients with DKD [46]. In addition, restriction of dietary protein and sodium may also reduce albuminuria and slow kidney function loss [47, 48].

Several medications influence the progression of DKD [22]. The blockade of renin-angiotensin system (RAS) attenuated the renal damage and albuminuria independently of their antihypertensive action [49]. However, long-term risk of GFR decline was not significantly different between persons with type 2 diabetes subjected to early treatment with losartan and those to placebo [50]. Additionally, among patients with advanced and progressive CKD (eGFR < 30 ml/min/1.73 m^2^) including diabetes subjects, the continuation of RAS inhibitors was not associated with a significant attenuation of the decrease in eGFR [51]. The use of sodium–glucose cotransporter 2 (SGLT2) inhibitors has been recommended in T2D to prevent progression of CKD in diabetes patients [52, 53] or even in patients without diabetes [54]. There is also an emerging body of evidence to suggest that glucagon-like peptide-1 receptor agonists (GLP-1RA) have actions to protect the function and structure of the kidney in models of diabetes. These actions are consistent with improved glucose control and significant weight loss. But SGLT2 inhibitors and GLP-1RAs may also have additive benefits such as improving inflammation and dysmetabolism [55]. Recently, finerenone, a new mineralocorticoid receptor antagonist (MRA), has proven to be effective in reducing kidney failure and kidney disease progression in patients with T2D with severely increased albuminuria and stage 3–4 CKD [56]. Previous data have supported a potential role of selective endothelin receptor antagonist atrasentan in protecting renal function in patients with T2D at high risk of developing end-stage kidney disease [57]. As compared with T2D group, more patients in DKD group take angiotensin-converting enzyme (ACE) inhibitor, angiotensin II receptor blocker (ARB) or SGLT2 inhibitor. We speculated that doctors are more likely to prescribe the above-mentioned drugs to diabetes patients with increased ACR and reduced kidney function. Few patients were prescribed GLP-1RA or finerenone in this study.

Our study has several strengths, especially the study design with two independent discovery and replication cohorts analyzed with standardized clinical evaluations. Few previous studies have addressed the association of urinary metabolites with DKD [16–18]. Instead, we used quality-controlled large-scale metabolomics profiling and discovered new associated urinary markers, which might facilitate the discrimination of DKD in the real-life clinical settings. There are several limitations conversely. Relatively small size of the cohorts and geographically close of the participants may limit the generalizability of this finding, so further studies with larger cohorts are needed to confirm our results. The observational study may not allow us to interpret the effect of metabolites in progression of DKD. The causality remains to be established through in vitro and animal studies as well as clinical trials although mediation analysis supported such hypothesis. Moreover, our findings should stimulate search for new therapeutic targets for prevention or treatment of DKD. Such treatments may include metabolite-based therapies to reduce urinary levels of up-regulated metabolites [58–61] or to neutralize the effects of up-regulated metabolites. Further studies are required to establish whether the exemplars or the other metabolites, or combinations of these metabolites offer the best therapeutic targets to slow or prevent DKD. Likewise, molecules in the inflammatory pathway for DKD deserve further study.

In conclusion, in patients with T2D, propionic acid, oxoadipic acid, leucine, isovaleric acid, isobutyric acid, indole-3-carboxylic acid, and SAH were potential biomarkers of DKD and performed well in discrimination of the presence of DKD. This study may pave the way for different precision medicine approaches in T2D, albeit with different in kidney involvement. Before been practicable in clinical settings, the DKD-associated metabolites require to be validated in larger cohorts. Further, it is necessary to investigate whether directing metabolism might reduce the risk of DKD in patients with T2D, particular their roles in inflammation, before a novel therapeutic approach can be implemented.

## Supplementary Information

Below is the link to the electronic supplementary material.

Supplementary file1 (TIF 1004 KB) Supplemental figure 1. Feature importance calculated by Boruta. The Boruta algorithm is a wrapper built around the random forest classification algorithm. It tries to capture all the important, interesting features might have in the dataset with respect to an outcome variable. Maximum iteration time is 1000. At every iteration, the algorithm compares the Z-scores of the shuffled copies of the features and the original features to see if the latter performed better than the former. If it does, the algorithm will mark the feature as important. In essence, the algorithm is trying to validate the importance of one feature by comparing with random shuffled copies, which increases the robustness. This is done by simply comparing the number of times a feature did better with the shadow features using a binomial distribution. A total of 19 Metabolites labeled as “Confirmed” (Green box) in the plot above can serve as biomarker for subsequent model building and prediction.

Supplementary file2 (TIF 2662 KB) Supplemental figure 2. Venn plot of potential biomarkers. Venn plot of 7 potential biomarkers from the 19 candidate biomarkers in discovery cohort and 31 differential metabolites of validation cohort was shown. Blue: 64 differential metabolites in the discovery cohort; Yellow: 31 differential metabolites in the totally independent validation cohort; Green: 19 candidate biomarkers in discovery cohort.

Supplementary file3 (XLS 294 KB) Supplemental Table 1. List of the absolute concentrations of 160 metabolites targeted measured using UPLC-MS/MS system in discovery cohort of 77 participants.

Supplementary file4 (XLS 396 KB) Supplemental Table 2. List of the absolute concentrations of 160 metabolites targeted measured using UPLC-MS/MS system in validation cohort of 115 participants.

Supplementary file5 (XLS 42 KB) Supplemental Table 3. List of the 64 differential metabolites match the threshold value in the discovery cohort. Threshold value for differential metabolite selection here is: (1) VIP > 1 in multi-dimensional statistics, and (2) *P* < 0.05 and |log_2_FC| >= 0 in univariate statistics.

Supplementary file6 (XLS 35 KB) Supplemental Table 4. List of the 31 differential metabolites match the threshold value in the validation cohort. Threshold value for differential metabolite selection here is: (1) VIP > 1 in multi-dimensional statistics, and (2) *P* < 0.05 and |log_2_FC| >= 0 in univariate statistics.

Supplementary file7 (DOCX 16 KB) Supplemental Table 5. IL-18 and CMI in the discovery and validation cohorts.

Supplementary file8 (DOCX 15 KB) Supplemental Table 6. Correlation between IL-18 and CMI in the pooled sample (*n* = 192; 92 DKD).

Supplementary file9 (DOCX 16 KB) Supplemental Table 7. Mediation analysis in the pooled sample.

## Data Availability

The datasets generated and analyzed in this study are available from the corresponding author upon reasonable request.
