# Supplementary figures and images for: Urinary metabolites associate with the presence of diabetic kidney disease in type 2 diabetes and mediate the effect of inflammation on kidney complication
Source: Acta Diabetol. 2023 May 15;60(9):1199–207. doi: 10.1007/s00592-023-02094-z (PMC10359369; doi:10.1007/s00592-023-02094-z)

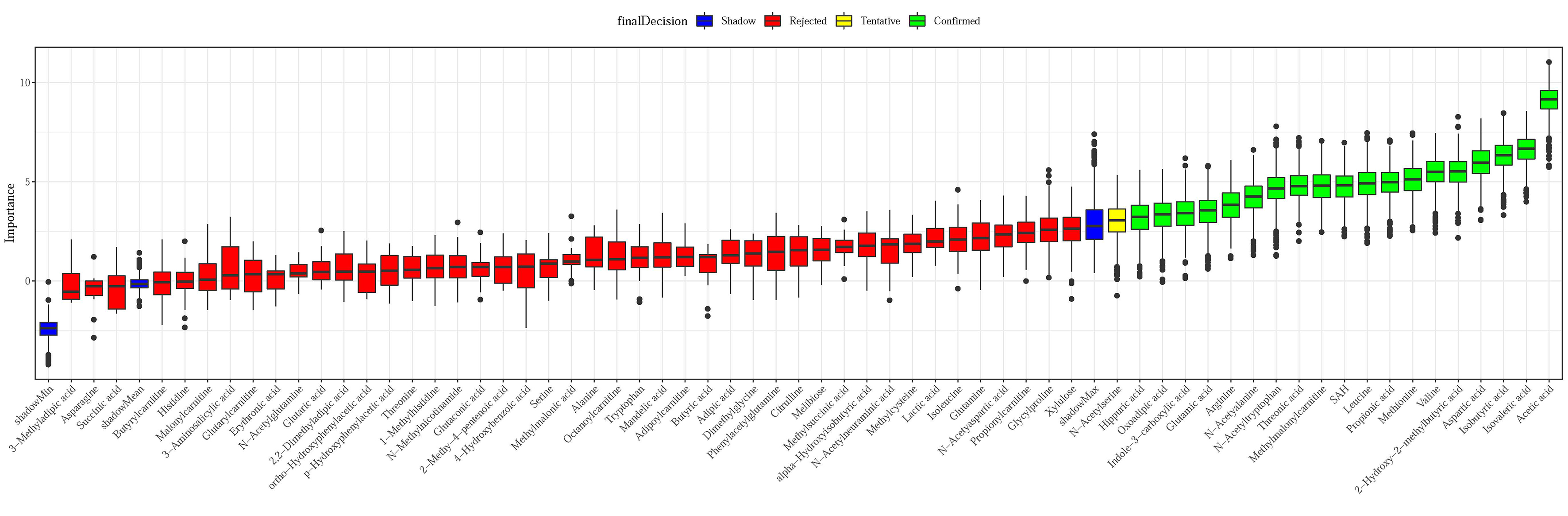

Supplement: Supplementary file 1 — Supplementary file1 (TIF 1004 KB) Supplemental figure 1. Feature importance calculated by Boruta. The Boruta algorithm is a wrapper built around the random forest classification algorithm. It tries to capture all the important, interesting features might have in the dataset with respect to an outcome variable. Maximum iteration time is 1000. At every iteration, the algorithm compares the Z-scores of the shuffled copies of the features and the original features to see if the latter performed better than the former. If it does, the algorithm will mark the feature as important. In essence, the algorithm is trying to validate the importance of one feature by comparing with random shuffled copies, which increases the robustness. This is done by simply comparing the number of times a feature did better with the shadow features using a binomial distribution. A total of 19 Metabolites labeled as “Confirmed” (Green box) in the plot above can serve as biomarker for subsequent model building and prediction. [file 592_2023_2094_MOESM1_ESM.tif]

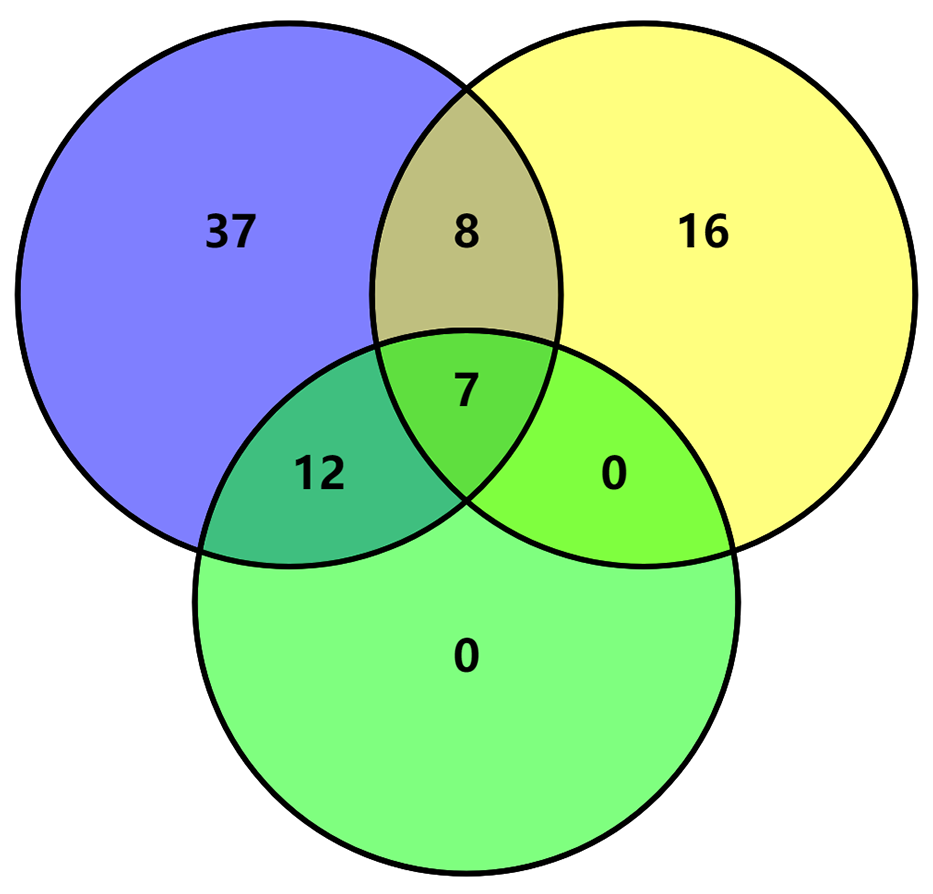

Supplement: Supplementary file 2 — Supplementary file2 (TIF 2662 KB) Supplemental figure 2. Venn plot of potential biomarkers. Venn plot of 7 potential biomarkers from the 19 candidate biomarkers in discovery cohort and 31 differential metabolites of validation cohort was shown. Blue: 64 differential metabolites in the discovery cohort; Yellow: 31 differential metabolites in the totally independent validation cohort; Green: 19 candidate biomarkers in discovery cohort. [file 592_2023_2094_MOESM2_ESM.tif]
