# Supplementary material for: Urinary metabolites associate with the presence of diabetic kidney disease in type 2 diabetes and mediate the effect of inflammation on kidney complication
Source: Acta Diabetol. 2023 May 15;60(9):1199–207. doi: 10.1007/s00592-023-02094-z (PMC10359369; doi:10.1007/s00592-023-02094-z)
Supplement: Supplementary file 7 — Supplementary file7 (DOCX 16 KB) Supplemental Table 5. IL-18 and CMI in the discovery and validation cohorts. [file 592_2023_2094_MOESM7_ESM.docx]

**Supplemental Table 4. IL-18 and CMI in the discovery and validation cohorts.**

|  | Discovery cohort | | | Validation cohort | | |
| --- | --- | --- | --- | --- | --- | --- |
|  | T2D | DKD | *P* value | T2D | DKD | *P* value |
| CMI | -0.85 (-1.33, -0.35) | 1.17 (-0.16, 2.75) | < 0.001 | -1.03 (-1.60, -0.19) | 0.28 (-0.53, 1.54) | < 0.001 |
| Serum IL-18 | 223.91 (163.63, 265.56) | 186.49 (141.31, 267.11) | 0.321 | 154.40 (101.89, 212.65) | 150.64 (109.15, 217.79) | 0.551 |
| Urinary IL-18 | 98.51 (78.09, 169.29) | 132.76 (103.44, 208.66) | 0.014 | 136.94 (94.02, 197.93) | 209.02 (110.62, 282.46) | 0.001 |

CMI was calculated as follows: 1.78925 × [isovaleric acid] + 0.770935 × [isobutyric acid] + 0.020945 × [leucine] + 0.966029 × [SAH] - 0.180627 × [propionic acid] + 0.176324 × [oxoadipic acid] + 0.210929 × [indole-3-carboxylic acid] - 2.866903.

CMI: composite index of 7 potential metabolite biomarkers; DKD, diabetic kidney disease; IL: interleukin; SAH, S-Adenosyl-L-homocysteine; T2D: type 2 diabetes.
