# Supplementary material for: Urinary metabolites associate with the presence of diabetic kidney disease in type 2 diabetes and mediate the effect of inflammation on kidney complication
Source: Acta Diabetol. 2023 May 15;60(9):1199–207. doi: 10.1007/s00592-023-02094-z (PMC10359369; doi:10.1007/s00592-023-02094-z)
Supplement: Supplementary file 8 — Supplementary file8 (DOCX 15 KB) Supplemental Table 6. Correlation between IL-18 and CMI in the pooled sample (n = 192; 92 DKD). [file 592_2023_2094_MOESM8_ESM.docx]

**Supplemental Table 5. Correlation between IL-18 and CMI in the pooled sample (n =192; 92 DKD).**

|  | β | *P* |
| --- | --- | --- |
| Serum IL-18 | 0.062 | 0.392 |
| Urinary IL-18 | 0.369 | < 0.001 |

Corrections are given as β.

CMI: composite index of 7 potential metabolite biomarkers; IL: interleukin.
