# Supplementary material for: Urinary metabolites associate with the presence of diabetic kidney disease in type 2 diabetes and mediate the effect of inflammation on kidney complication
Source: Acta Diabetol. 2023 May 15;60(9):1199–207. doi: 10.1007/s00592-023-02094-z (PMC10359369; doi:10.1007/s00592-023-02094-z)
Supplement: Supplementary file 9 — Supplementary file9 (DOCX 16 KB) Supplemental Table 7. Mediation analysis in the pooled sample. [file 592_2023_2094_MOESM9_ESM.docx]

**Supplemental Table 6. Mediation analysis in the pooled sample.**

|  | β | *P* | 95% CI | |
| --- | --- | --- | --- | --- |
| a: Effect of urinary IL-18 on CMI | 2.281 | 0.0001 | 1.231 | 3.276 |
| b: Effect of CMI on DKD | 1.415 | < 0.001 | 1.114 | 2.444 |
| c’: Direct effect of urinary IL-18 on DKD | 1.062 | 0.9658 | -1.727 | 4.061 |
| a×b: Indirect effect of urinary IL-18 on DKD | 3.227 | < 0.05 | 1.818 | 6.577 |
| c = a×b+c’: Total effect of urinary IL-18 on DKD | 4.288 | - | - | - |
| Mediation effect = b/c×100% | 32.99% | - | - | - |

Mediation analysis was carried out in a fully adjusted model, comprising age, gender, hypertension, diabetes duration, BMI, HbA1c, total cholesterol, albumin, and eGFR. β = standardized coefficient of regression.

CI: confident intervals; CMI: composite index of 7 potential metabolite biomarkers; DKD, diabetic kidney disease; IL: interleukin.
